# Molecular Chaperone Accumulation in Cancer and Decrease in Alzheimer's Disease: The Potential Roles of HSF1

**DOI:** 10.3389/fnins.2017.00192

**Published:** 2017-04-21

**Authors:** Stuart K. Calderwood, Ayesha Murshid

**Affiliations:** Molecular and Cellular Radiation Oncology, Beth Israel Deaconess Medical Center, Center for Life Sciences 610, Harvard Medical SchoolBoston, MA, USA

**Keywords:** molecular chaperone, heat shock protein, cancer, Alzheimer's disease, proteotoxic stress

## Abstract

Molecular chaperones are required to maintain the proteome in a folded and functional state. When challenges to intracellular folding occur, the heat shock response is triggered, leading to increased synthesis of a class of inducible chaperones known as heat shock proteins (HSP). Although HSP synthesis is known to undergo a general decline in most cells with aging, the extent of this process varies quite markedly in some of the diseases associated with advanced age. In Alzheimer's disease (AD), a prevalent protein folding disorder in the brain, the heat shock response of some critical classes of neurons becomes reduced. The resulting decline in HSP expression may be a consequence of the general enfeeblement of many aspects of cell physiology with aging and/or a response to the pathological changes in metabolism observed specifically in AD. Cancer cells, in contrast to normal aging cells, undergo *de novo* increases in HSP levels. This expansion in HSP expression has been attributed to increases in folding demand in cancer or to the evolution of new mechanisms for induction of the heat shock response in rapidly adapting cancer cells. As the predominant pathway for regulation of HSP synthesis involves transcription factor HSF1, it has been suggested that dysregulation of this factor may play a decisive role in the development of each disease. We will discuss what is known of the mechanisms of HSF1 regulation in regard to the HSP dysregulation seen in in AD and cancer.

## Introduction

Cancer and Alzheimer's disease (AD) each affect large proportions of the population and, at least in their sporadic forms are much more prevalent in older people. In both diseases, there is strong evidence for loss of regulation of molecular chaperone function that may contribute to the morbidity of the diseases (Calderwood et al., 2009; Calderwood and Gong, 2016). However, these changes take different forms in the two disease types. In short, cancer is associated with expansion in molecular chaperone expression, while onset of AD and other neurodegenerative diseases has been associated with reduced HSP levels and a decrease in the ability to deal with proteotoxic stress (Batulan et al., 2003; Calderwood and Gong, 2016). These findings are confluent with numerous epidemiological studies that show a negative correlation between the risk of cancer in persons with AD or other neurodegenerative diseases (Roe et al., 2005; Driver, 2014). We will explore here regulation of molecular chaperone synthesis and how it may become modified during development of AD and cancer.

## Heat shock proteins- an inducible class of molecular chaperones

The most intensely studied property of HSPs is their facilitation of the pathways of protein folding (Lindquist and Craig, 1988; Kayser et al., 2013; Kityk et al., 2015). They thus belong to the families of molecular chaperones. HSPs possess the capacity to recognize structures commonly found in the interior of proteins and to bind such structures. Their major role appears to deter the formation of quasi-stable conformations during folding (Ellis, 2007). With this aid, nascent proteins or polypeptides being rescued from denaturation will then collapse into their low-energy, native conformations in which they can direct cell metabolism. In order to be released from client proteins after folding, and take part in further rounds of activity, Hsp70 and other chaperones utilizes an intrinsic ATPase domain to hydrolyze ATP and assume a free conformation (Kityk et al., 2015). The low molecular weight chaperone Hsp27, lacking an ATPase domain, requires rescue by Hsp70 to release from its clients. Hsp27, Hsp70, and Hsp90 can act in relay to permit unfolded proteins to achieve functional activity in a stepwise manner (Calderwood and Gong, 2016). Although there are a number of other HSP families, we have concentrated on Hsp27, Hsp70, and Hsp90, as they are the most intensely studied HSPs in cancer and AD. In some cases, proteins with fragile conformations remain associated with Hsp90 in order to maintain a functional conformation (Kirschke et al., 2014). Thus, most cells contain high concentrations of these proteins, particularly Hsp90 to chaperone the proteome. During proteotoxic stress, a subclass of inducible chaperones is induced at the transcriptional level to boost chaperoning capacity, and these are the HSPs (Richter et al., 2010). For a more detailed overview of molecular chaperone function and interaction with protein co-factors (co-chaperones), readers are referred to a previous review (Calderwood, 2013).

## Regulation of the heat shock response by HSF1

**Figure 1:**
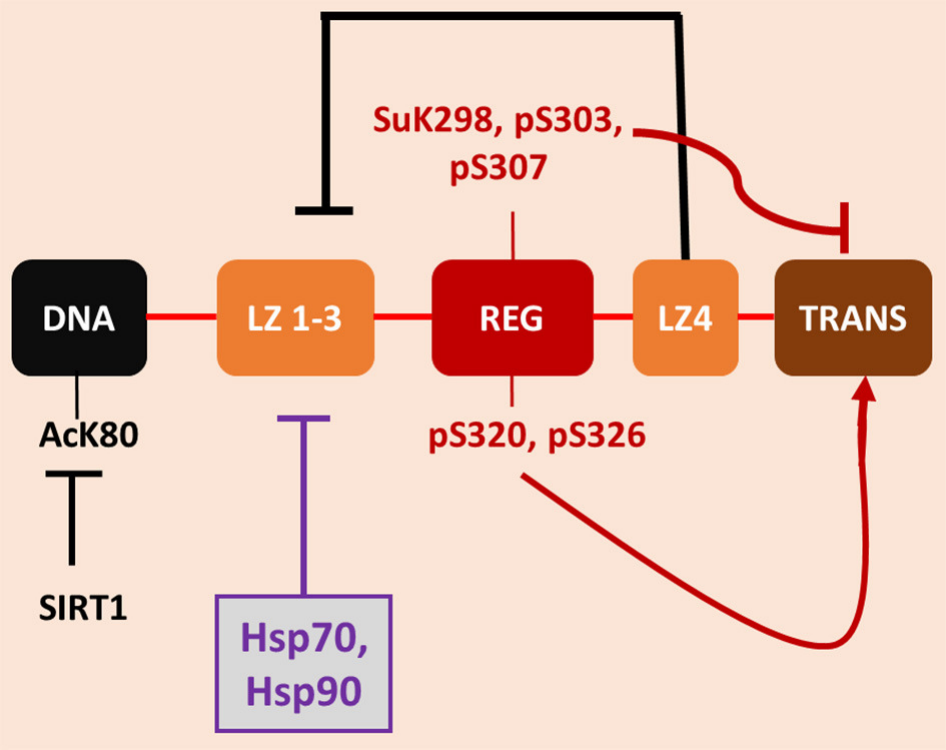
**Regulatory mechanisms governing HSF1 activity**. The figure depicts the major functional domains within HSF1 in their linear organization along the protein structure. (note the domains are not drawn to scale). There are, N terminally to C-terminally, a DNA binding domain (DNA), a trimerization domain (leucine zipper 1-3 or LZ1-3), a central regulatory domain (REG), a fourth region of leucine zipper or hydrophobic heptad repeat sequence (LZ4) and the C-terminal double *trans*-activation domains (TRANS). The primary mechanism for HSF1 regulation appears to the intramolecular coiled coil interaction between LZ4 and LZ 1-3 that prevent trimerization and DNA binding under basal conditions, which is severed during heat shock. A second regulatory mechanism is feedback repression exerted by Hsp70 and Hsp90 that can bind at various positions in the HSF1 molecule to effect inhibition. The regulatory domain contains an array of phosphorylation sites that can govern activity. Most notable among these is serine 303 (pS303) whose phosphorylation mediates inhibition of HSF1, and sumoylated lysine 298 (SuK298). In addition, other PTMs have been found elsewhere in the molecule, with lysine 80 (K80) undergoing repressive acetylation (AcK80) that can be relieved by the deacetylase sirtuin 1.

Exposure of almost any cell to heat shock leads to the almost immediate transcription, translation and accumulation of a cohort of HSPs that increase to quite remarkable levels when the stress is pronounced (Richter et al., 2010). Such cells then become resistant to further stress by heat shock (Li and Hahn, 1980). We now know that a major property of HSPs is to facilitate the folding of a large proportion of intracellular proteins toward functional conformations (Ellis, 2007). Heat shock triggers the unfolding and aggregation of many intracellular proteins—hence the development of the heat shock response early in evolution to cope with environmental stress (Zhang and Calderwood, 2011). The key effector of *HSP* gene transcription is heat shock factor 1 (HSF1), a sequence specific factor that binds upstream of all *HSP* genes at the onset of stress (Wu, 1995). There is currently no unique hypothesis as to the mechanism by which HSF1 senses the heat shock and becomes activated. HSF1 may be able to both sense the change in temperature shift directly, as with a thermometer, or may have evolved to respond to the toxic effects of the heat shock such as protein unfolding within the cytoplasm (Zhong et al., 1998; Zou et al., 1998). In all eukaryotes, HSF1 responds to stress by undergoing a monomer to trimer transition and becomes heavily phosphorylated, leading to its acquiring ability to rapidly bind to DNA and activate transcription (Akerfelt et al., 2010). It became clear quite early that transcriptional activation by mammalian HSF1 required more than its trimerization and DNA binding (Price and Calderwood, 1991). Recent studies also suggested a similar two stage activation in yeast (Zheng et al., 2016). In fact, some stimuli- such as exposure to high levels of sodium salicylate could lead to almost quantitative HSF1 binding to DNA without increasing HSP transcription (Jurivich et al., 1992; Housby et al., 1999). Heat shock sensitivity of HSF1 would seem to require at least two regulatory events. The Voellmy lab were able to recreate HSF1 activation in the absence of heat shock by combining: (1) sodium salicylate stimulation (which produces trimerization and DNA binding) with (2) exposure to phosphatase inhibitors- strongly suggesting that the second stimulus for HSF1 induction involves direct or indirect effects of phosphorylation (Voellmy, 1994; Figure 1).

Work from the Kingston lab next provided a structural basis for the two-stimulus hypothesis by the discovery of a new temperature sensitive domain in HSF1 remote from the trimerization domain (Green et al., 1995; Newton et al., 1996). This region—called the regulatory domain was shown to be in the middle of the HSF1 protein sequence, C-terminal to the trimerization and DNA binding domains and upstream from the *trans*-activation domains and to control HSP gene transcription independently from DNA binding. Interestingly, the regulatory domain appeared to repress *trans* activation in the absence of stress but transmitted the inducing effects of heat shock to the trans activation domains (Green et al., 1995; Newton et al., 1996). Interestingly, this domain contains both positively acting and repressive phosphorylation sites (Calderwood et al., 2010). Serine 303 is phosphorylated by glycogen synthase kinase 3 (GSK3) under non-stress conditions and represses HSF1 through mechanisms including induction of repressive SUMO modifications as well as nuclear export (Wang et al., 2003; Anckar et al., 2006). Heat shock overrides this form of repression as well as leading to activation of positively acting phosphorylation on serines 320 and 326 (Zhang et al., 2011; Chou et al., 2012). One could thus envisage a scenario in which stress triggered the first activating stimulus, leading to trimerization and rapid localization of HSF1 on *HSP* genes, and in a similar time frame activated a second signal through the regulatory domain to render the chromatin-bound HSF1 able to positively regulate *trans*-activation. Many questions still remain of course- such as the nature mechanism by which the regulatory domain led to the induction of the remote *trans*-activation domains and whether the activities of the trimerization region and the regulatory domain were in some way coordinated. In addition, there are important PTMs outside the regulatory domain. Serine 121 is another repressive phosphorylation site that acts to suppress DNA binding (Wang et al., 2006). In addition, HSF1 is modified by acetylation, most notably at lysine 80, an effect that reduces the duration of DNA binding (Westerheide et al., 2009). HSF1 activation can be effected by a range of deacetylases, including sirtuin 1, HDAC7 and HDAC9 that each remove the acetyl group from K80 and increase the lifetime of association of HSF1 trimers with DNA (Westerheide et al., 2009, 2012; Zelin and Freeman, 2015). It is of note that histone acetylase p300 is recruited by HSF1 to heat shock genes along with positive transcriptional elongation factor b (pTEFb) and is involved in *trans* activation by acetylating key histones (Zhang et al., 2011). This event could also be involved in HSF1 switch off, in the resolution of the response through K80 acetylation.

However, at some time within the aging organism, these complex regulatory mechanisms go wrong and the heat shock response loses its ability to switch on or off at the appropriate moment, effects that may differ in different organs and different diseases (Calderwood et al., 2009). The multiple molecular inputs into HSF1 regulation may each play distinct roles in the dysregulation of the factor in disease. It is also apparent that different triggers may activate HSF1 by alternative mechanisms. Fast activation in heat shock may involve the intrinsic capacity to sense temperature, while more gradual triggers as in cancer may be mediated through reversal of repressive pathways and activating PTMs (Zhong et al., 1998; Khaleque et al., 2005).

## HSPs and the promotion of cancer

It is now accepted that the levels of HSPs are relatively high in most types of human cancer compared to their normal tissues of origin (Ciocca and Calderwood, 2005; Ciocca et al., 2013). Most such studies have concerned themselves with a select group of HSPs—Hsp27, Hsp70, and hsp90 - and we will concentrate mostly on these proteins in this discussion (Calderwood and Gong, 2016). The proteins shown to play causal roles in carcinogenesis are by definition effector proteins in the processes that define malignancy, such as growth factor independent growth, escape from cell death and senescence pathways, promotion of angiogenesis and metastasis (Hanahan and Weinberg, 2011). It is evident that the canonical functions of HSPs do not appear to include the ability to directly effect these properties (Ellis, 2007). Their best-known properties exclusively involve protein folding, as described above. However, HSPs facilitate the properties that give cancer its morbidity (Calderwood and Gong, 2016). Indeed, HSPs are required for stimulus-independent growth, escape from apoptosis and senescence, angiogenesis, invasion and metastasis (Garrido et al., 2006; Yaglom et al., 2007; Thuringer et al., 2013; Calderwood and Gong, 2016). Many of these effects involve the chaperoning of oncoproteins involved in each of these cell behaviors. Interestingly, HSPs may contribute in different ways to transformation and tumor progression, depending on the driver oncogene that mediates tumorigenesis. For instance, knockout of Hsp70 reduced the growth of mammary cancers transformed by the oncogene Her2 due to cell senescence (Meng et al., 2011). However, in mammary epithelial cells transformed by the Polyoma Middle T antigen, the effects of hsp70 inactivation was to reduce tumor cell invasion and metastasis (Gong et al., 2015). Although an increasing role for HSPs in cancer is emerging, much needs to be learned regarding their multiple roles in disease progression.

**Figure 2:**
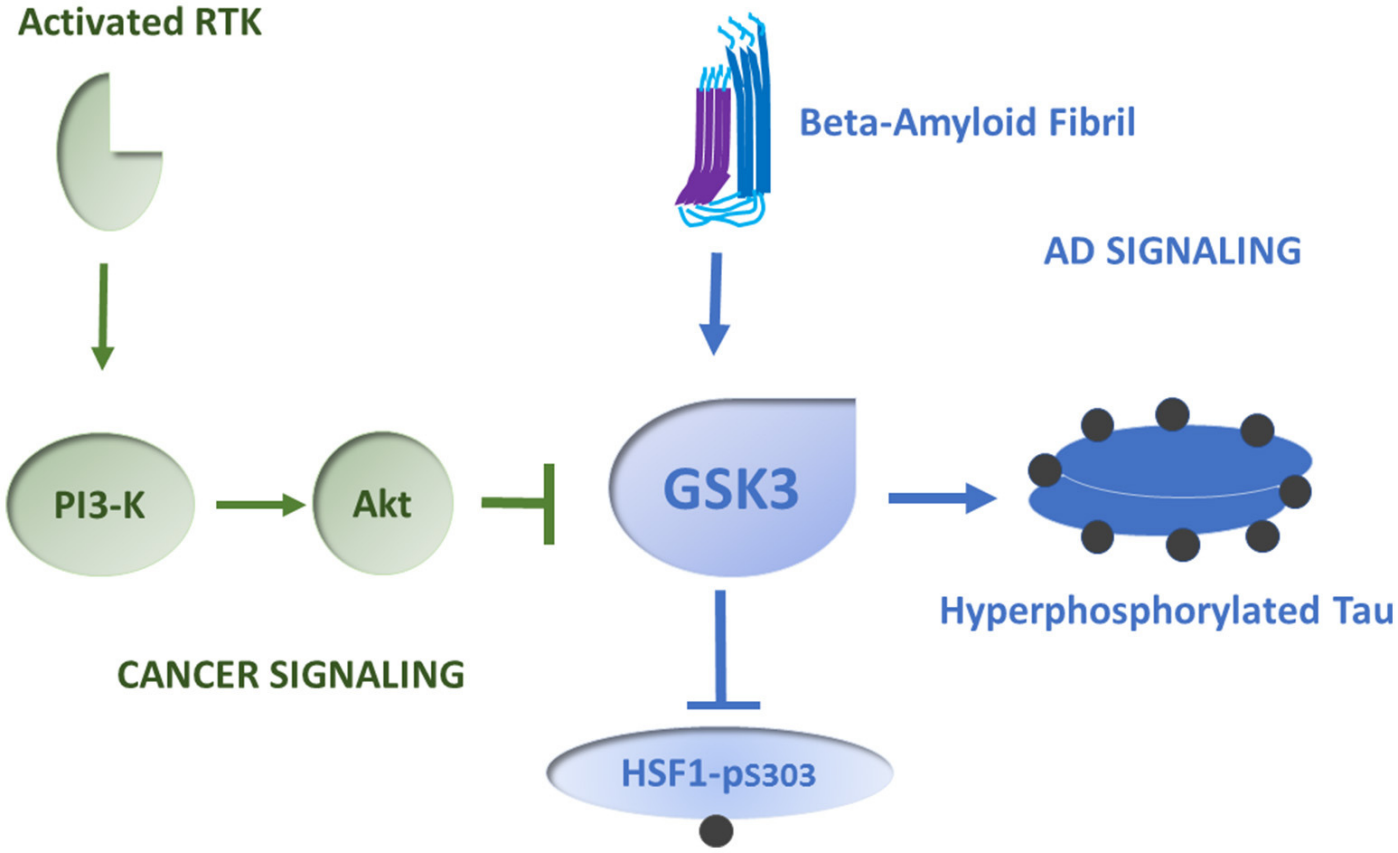
**Role of GSK3 in HSF1 regulation in cancer and AD**. We depict GSK3 as a key HSF1 repressor that may govern its activity in cancer and AD. In many types of cancer, GSK3 becomes inhibited when the enzyme Akt is induced by a cascade response involving activated receptor tyrosine kinases (RTK), and phosphatidylinositol-3-kinase (PI-3K). Akt phosphorylates and inactivates GSK3. Thus, HSF1 is relieved of repression and can induce HSPs in tumors. In AD, GSK3 shows increased levels of activity, leading to HSF1 repression. This mechanism may involve activation of GSK3 downstream of aggregated amyloid beta fibrils. Concomitantly Tau is hyperphosphorylated by a mechanism involving active GSK3, leading to Tau aggregation. Phosphorylated residues on HSF1 and Tau are suggested by dark spheres.

One hypothesis that has arisen to explain the aberrantly high concentrations of HSPs in cancer is known as “addicted to chaperones.” Tumor cells are thought to be in some ways similar to mildly heat shocked cells, with oncogene overexpression and mutation, polyploidy and exaggerated levels of translation generating an intracellular folding demand (Workman et al., 2007). This hypothesis draws heavily upon the proposed mechanism for HSF1 activation involving reversal of its feedback inhibition by HSPs such as Hsp90 and Hsp70 (Zou et al., 1998; Gómez et al., 2008; Figure 1). Such HSPs are thought to act by suppressing HSF1 trimerization or recruiting co-repressors to HSF1, while activation involves the sequestration of the HSPs in protein aggregates and release of free HSF1 to pursue its transcriptional role (Zou et al., 1998). Hsp90 targeted drugs indeed have the predicted property of causing the degradation of unchaperoned oncoproteins while activating HSF1 and HSP synthesis (Workman et al., 2007; Conde et al., 2009). However, this hypothesis is difficult to prove conclusively and is not universally supported; it is not clear that the tumor environment can be shown to be in a state of folding demand (Colvin et al., 2014). It also seems apparent that that HSF1 can be coupled directly to some of the cancer signaling pathways. In cancer, signal transduction pathways that are normally coupled tightly to the occupation of growth receptors can operate independently, due to mutations or increases in expression in signaling intermediates (Hanahan and Weinberg, 2011). As mentioned above, HSF1 is repressed by phosphorylation on serine 303, within the regulatory domain, through the kinase GSK3 (Chu et al., 1996). In breast cancer HSF1 repression can be relieved by exposure to the ligand heregulin that binds cell surface receptor tyrosine kinase HER3. HER3 activation then activates HSF1 through its triggering of the kinase Akt that can in turn lead to inhibition of GSK3 and relief of repression (Khaleque et al., 2005). Feedback regulation through HSPs does not seem to be involved in this case and HSP synthesis and treatment resistance can thus be coupled directly to mammary cancer signaling pathways known to promote malignancy. Phosphorylation at an adjacent domain, serine 326 causes HSF1 activation (Chou et al., 2012). It is notable that HSF1 in mammary cancer stem cells, the cells that govern tumorigenicity, invasion and metastasis, is constitutively phosphorylated on serine 326 and dephosphorylated on serine 303, suggesting a causal role for activated HSF1 in stemness (Chou et al., 2015; Gong et al., 2015). Indeed, activated HSF1 induces the stem cell renewal factor beta-catenin (Chou et al., 2015). Thus, HSF1 activation and HSP expression in cancer may involve relief of the repressive effects of both HSPs and GSK3 as well as positive input through serine 326 (Figures 1, 2). This mechanism appears to resemble the two-signal model of HSF1 activation by heat shock developed by Voellmy et al and others, with HSP sequestration permitting step (1), HSF1 escape from chaperone repression and trimerization and (2) phosphorylation in the regulatory domain providing the second signal for activation (Voellmy, 1994; Calderwood et al., 2010). It is also notable that HSF1 can exert tumorigenic effects through non-HSP chromosomal targets, including metastasis associated protein 1 and others (Khaleque et al., 2008). Therefore, HSF1 regulates both HSP expression as well as non-HSP targets that may go some way to explaining its potency in carcinogenesis (Ciocca et al., 2013).

## Decline of the heat shock response in Alzheimer's disease

It is now widely accepted that Alzheimer's disease (AD) and other neurodegenerative diseases are, at least partially, protein folding disorders (López-Otin et al., 2013; Cardinale et al., 2014). In this review, we have concentrated on three chaperones-Hsp27, Hsp70, and Hsp90 in cancer and AD. The rationale for this choice was that the vast majority of publications regarding the roles of HSPs in cancer deal with these three chaperones and we thus concentered on these when comparing the diseases (Ciocca and Calderwood, 2005; Calderwood and Gong, 2016). However, readers are referred to a recent publication showing a complex pattern of multiple chaperones and co-chaperones that are modulated over time in age-dependent neurodegenerative disease- some of which are increased while others decline (Brehme et al., 2014). Two of the major lesions associated with AD have been identified as large protein aggregates, known as senile plaques that accumulate in the cerebral cortex during disease progression, leading to loss of neurons and synapses and ultimately dementia. These two lesions are: (1) Beta amyloid plaques, derived from the amyloid precursor protein APP in the neuronal plasma membrane. Peptides derived from APP of 39-43 amino acids spontaneously form oligomers and ultimately highly insoluble myeloid fibrils (Sipe and Cohen, 2000). Such beta amyloid fibrils are toxic to adjacent neurons due to their disruption of calcium homeostasis, leading to apoptosis. (2) AD also belongs to the tauopathy family of diseases, each characterized by progressive formation of tangled aggregates containing the microtubule stabilizing protein Tau (Holmes et al., 2014). The mechanisms of Tau aggregation are not fully understood, but involve its hyperphosphorylation by GSK3. Hyperphosphorylated Tau can then recruit healthy, normally phosphorylated Tau into aggregates and tangles (Alonso et al., 1996; Wang et al., 2013). Tau aggregation is toxic to cells, leading to the disruption of microtubules essential for molecular transport along axons, a key aspect of neuronal survival.

HSPs have been shown to be involved in the healthy processing of amyloid beta, which can be found associated with Hsp10, Hsp27, Hsp60, Hsp70, and Hsp90 (Maiti et al., 2014). Although it is unlikely that intracellular chaperones could directly interact with extracellular amyloid beta plaques, they may influence the chaperoning of APP in neurons (Maiti et al., 2014) or they could influence the proteolytic processing of amyloid plaques phagocytosed by brain resident macrophages. Hsp70 binds to Tau reducing its hyperphosphorylation, decreasing aggregation and promoting Tau binding to microtubules (Sarkar et al., 2008). Hsp90 plays a similar role in Tau homeostasis (Sarkar et al., 2008). Degradation of Tau was observed after treatment with Tau-derived synthetic peptides and appeared to involve the substitution of Hsp70 associated with tau oligomers by Hsp90 (Thompson et al., 2012). Hsp90 rather than Hsp70 appeared to play a primary role in Tau degradation through the proteasome (Thompson et al., 2012). In addition, Hsp27 was shown to rescue neuronal deficits in *tau* knockout mice. These effects of Hsp27 appeared to involve its phosphorylation-dependent chaperoning capacity, as mutation of the key phosphorylation sites in the chaperone ablated its ability to rescue the *tau* transgenics (Abisambra et al., 2010). Each HSP family member then can participate in the deterrence of AD lesions in cells or rodent models of the disease.

It is notable that the most common neurodegenerative diseases: AD, Huntington's disease and Parkinson's disease, each associated with protein folding disorder, occur as the organism ages (Calderwood et al., 2009). In addition, although each disease type involves distinct proteins with dominantly aggregating properties (respectively—Tau, huntingtin, alpha-synuclein), neurodegenerative symptoms develop with aging in each disease (Calderwood et al., 2009). This time-dependent window for induction of neurodegenerative diseases has been attributed to a decline in the proteotoxic stress response with age, permitting the phenotype of the dominantly aggregating proteins to become revealed (Sherman and Goldberg, 2001; Hands et al., 2008; Winklhofer et al., 2008).

## HSP regulation in the brain and ensuing dysregulation in AD

There appears to be an overall decline in protein quality control pathways and HSP synthesis with aging in many tissues, including the brain, muscle and liver, as reviewed in Calderwood et al. (2009). Studies in invertebrates have identified HSF1 as a significant longevity factor and, for instance this factor plays a key role in the enhanced longevity mediated by dietary restriction and its inactivation reduces lifespan in *C. elegans* (Hsu et al., 2003; Steinkraus et al., 2008). HSF1 mediated chaperone synthesis may thus promote longevity by maintaining protein folding capacity (Hsu et al., 2003). It has been shown that HSF1 is of limited activity in neuronal cells in tissue culture, suggesting that these tissues may be critically sensitive to age-dependent declines in HSP inducibility and ability to respond to folding deficits (Batulan et al., 2003). Interestingly these effects appear to be mediated by the regulatory domain of HSF1 (Figure 1) and deletion of this region led to renewed ability of the factor to promote transcription in a neuronal cell setting (Batulan et al., 2003). Interestingly the regulatory domain contains a repressive phosphorylation site for GSK3 and removal of this site would lead to relief of repression (Figure 2). GSK3 is increased in activity in the aging brain and is of critical importance in AD, as this is the principal kinase that leads to Tau phosphorylation and tangle formation (Hooper et al., 2008). Thus, elevated GSK3 activity may offer double jeopardy for AD, in promoting Tau pathology while repressing HSF1 and the heat shock response. In both HSF1 and Tau, GSK3 phosphorylation is associated with recruitment of 14-3-3 adapter proteins that leads in the case of HSF1 to transcriptional repression and in Tau to increased fibril formation (Wang et al., 2003; Qureshi et al., 2013). Another kinase that impacts HSF1 is mTOR (Chou et al., 2012). It was shown that, although mTOR can activate HSF1, increased HSF1 activity seemed to lead, conversely to decreases in mTOR signaling (Bandhakavi et al., 2008). Increases in mTOR activity are regarded as important in AD pathology and HSF1 may thus be important in protection against such mTOR-mediated morbidity (Wang C. et al., 2014). In addition, HSF1, Hsp60, Hsp70, and Hsp90 were each expressed at low levels in the cerebella of AD rats (Jiang et al., 2013). Overexpression of HSF1 was shown to increase HSP levels in the cerebellum and lead to an increase in the number of Purkinje cell bodies in the brains of mouse models of AD (Jiang et al., 2013). Another key HSF1 regulator is the histone deacetylase sirtuin 1, a key longevity factor which deacetylates HSF1 and increases its binding to HSP promoters (Figure 1). Sirtuin 1 mRNA and protein levels are known to be reduced in the brains of AD patients concomitantly with Tau accumulation, suggesting a further route to HSF1 malfunction in AD (Julien et al., 2009). Another mechanism involved in loss of HSF1 activity could be increased HSF1 degradation due to the ubiquitin E3 ligase NEDD4. Degradation of HSF1 through the NEDD4 pathway was antagonized when HSF1 was deacetylated by sirtuin1 (Kim et al., 2016). Aggregated alpha-synuclein was shown to trigger this pathway targeting HSF1 in transfected neuroblastoma cells. It might be rewarding to study the potential role of this pathway in tauopathies such as AD, particularly in light of the known reduction in Sirt1 levels in clinical AD. (Julien et al., 2009). HSF1 is also known to have non-HSP transcriptional targets and these may be important in AD (Khaleque et al., 2008). HSF1 activates transcription of transthyretin (TTR) a protein that can impact symptoms in Mouse AD model by inhibiting beta-amyloid aggregation and detoxifying the amyloid oligomers (Wang X. et al., 2014).

All in all then, the balance of evidence suggests that the activity of HSF1 and the levels of HSPs are depleted in AD. It would however, be desirable to obtain a fuller picture of dysregulation of the heat shock response in the AD brain.

## Discussion- comparing the reciprocal dysregulation of the heat shock response in cancer and AD

Although cancer and AD are diseases encountered later in life, HSP metabolism is altered in different directions in each case. These findings are consonant with epidemiological studies showing a negative correlation between the risk of cancer in persons with AD (Roe et al., 2005; Driver, 2014). These differences may in some ways reflect the gulf between terminally differentiated neurons in AD brains, accumulating a lifetime's damage to DNA and proteins, and de-differentiating, evolving cancer cells that maintain the ability to proliferate and renew. However, in each case there appears to be an acquired folding deficit, with increases in unfolded and aggregated proteins (Sherman and Goldberg, 2001; Winklhofer et al., 2008; Calderwood and Gong, 2016). Despite this, HSP levels respond by increasing in cancer and declining in AD. Thus, the simple hypothesis of HSF1 induction due to loss of feedback repression by Hsp70 or Hsp90 does not help us with understanding the changes in chaperone expression encountered in AD. One potential unifying thread explaining some of the differences in cancer and AD might be the kinase GSK3, the repressor of HSF1 (Chu et al., 1998; Figure 2). In malignant diseases, many tumorigenic pathways, such as those conferred by oncogenic receptors PDGF-R, EGF-R, and HER2, by widespread increases in PI-3 kinase and loss of the phosphatase PTEN often encountered in cancer, converge on the kinase Akt that is a GSK3 repressor (Yuan and Cantley, 2008; Chalhoub and Baker, 2009). Indeed, HSF1 can be activated directly in mammary cancer through the Her2 pathway by activated Akt which mediates GSK3 inhibition (Khaleque et al., 2005). Highly malignant mammary cancer stem cells contain very low levels of the GSK3 target HSF1-phospho-S303, consonant with the findings of that levels of Hsp70 are required for stemness (Chou et al., 2015). GSK3 is also intimately involved in the pathology of AD, with increases in GSK3 activity contributing to the multiple areas of neuronal pathology associated with the disease (Hooper et al., 2008; Kremer et al., 2011). GSK3 plays the key role in Tau phosphorylation and aggregation and may be involved in coupling the APP / beta amyloid pathway to Tau pathology (Kremer et al., 2011; Figure 2).

The pharmacological targeting of HSP activity in each disease is currently under investigation. In cancer, Hsp90 inhibitors have been extensively tested as therapeutics, although problems with normal tissue toxicity currently limit their clinical application (Workman et al., 2007). There is also interest in targeting HSP interactions with Tau in AD, and both Hsp70 and Hsp90 have recently been investigated (Jinwal et al., 2010; Thompson et al., 2012). This approach may ultimately have considerable promise for each disease, although it is currently limited by the toxicity to healthy tissues that may accrue when whole families of chaperones are targeted by inhibitors. Drugs specific for individual members of the HSP families would offer the promise of specificity and sparing of healthy tissues, although this approach may also be conceptually difficult due to the high levels of conservation among the chaperone family members (Lindquist and Craig, 1988).

## Author contributions

All authors listed, have made substantial, direct and intellectual contribution to the work, and approved it for publication.

## Funding

This work was supported by funding from NIH research grants: R01CA119045, RO1CA47407 and RO1CA176326.

### Conflict of interest statement

The authors declare that the research was conducted in the absence of any commercial or financial relationships that could be construed as a potential conflict of interest.
